# *Astragalus membranaceus*: A Traditional Chinese Medicine with Multifaceted Impacts on Breast Cancer Treatment

**DOI:** 10.3390/biom14101339

**Published:** 2024-10-21

**Authors:** Zhong Tang, Xuefei Tian

**Affiliations:** 1 School of Integrated Chinese and Western Medicine, Hunan University of Chinese Medicine, Changsha 410208, China; tangzhong@hnucm.edu.cn; 2 Department of Galactophore, The First Hospital of Hunan University of Chinese Medicine, Changsha 410007, China; 3 Hunan Key Laboratory of Traditional Chinese Medicine Prescription and Syndromes Translational Medicine, Hunan University of Chinese Medicine, Changsha 410208, China; 4 Key Laboratory of Oncology of Traditional Chinese Medicine, Hunan University of Chinese Medicine, Changsha 410012, China; 5 Key Laboratory of Traditional Chinese Medicine for Mechanism of Tumor Prevention and Treatment, Hunan University of Chinese Medicine, Changsha 410208, China

**Keywords:** breast cancer, *Astragalus membranaceus*, antitumor, combined therapy, chemical compounds

## Abstract

Breast cancer, the most prevalent malignant tumor among women globally, remains a critical area of focus for researchers striving to refine therapeutic approaches. As an important component of traditional Chinese medicine, *Astragalus membranaceus* (AM) has demonstrated potential for multifaceted impacts on breast cancer treatment through various mechanisms. To guide clinical practice and further explore the under-researched field of AM in breast cancer treatment, this paper mainly reviews the regulatory roles of AM-derived compounds and extracts on breast cancer cell proliferation, migration, invasion, and chemoresistance. Furthermore, this study delves into the synergistic effects observed when AM is co-administered with chemotherapeutic agents, including the enhancement of chemosensitivity, mitigation of toxic side effects, and reversal of drug resistance. This review indicates that AM holds promise not only as a therapy in breast cancer treatment but also paves the way for innovative integrated treatment approaches that combine the benefits of traditional medicine with modern pharmaceuticals. Nevertheless, future research endeavors are also urged to elucidate the in vivo pharmacological effects and underlying mechanisms of AM to inform more effective clinical treatment strategies.

## 1. Introduction

Breast cancer is the most common cancer among women. According to the latest GLOBOCAN estimates released by the International Agency for Research on Cancer, breast cancer in women accounts for 11.6% of all cancers globally and 6.9% of all cancer-related mortalities [1]. For patients with breast cancer that has not spread to other parts of the body, surgical intervention remains the most effective treatment strategy [2], with specific surgical procedures selected based on different objectives [3]. Radiotherapy is also an effective means of treating breast cancer, typically used as an adjunct to surgical treatment to further reduce the rate of cancer metastasis [4] and recurrence [5]. For patients with metastatic breast cancer, systemic pharmacological therapy is the primary approach. The pharmacological treatment of breast cancer includes chemotherapy, targeted therapies, immunotherapeutic drugs, and endocrine therapy agents. Additionally, traditional Chinese medicine, gene therapy, and stem cell treatments have also shown positive effects in the treatment of breast cancer [6]. Despite significant advancements in the treatment of breast cancer, issues such as drug resistance in cancer cells and severe side effects post-treatment remain unresolved.

*Astragalus*, a genus within the *Fabaceae* family, is one of the largest genera and is widely distributed across the Northern Hemisphere, South America, and Africa, having a broad spectrum of pharmacological effects. *Astragalus membranaceus* (AM), one of the most frequently used traditional Chinese medicines, is renowned for its diverse pharmacological effects, including immune stimulation [7,8,9], antioxidant properties [10], blood sugar reduction [11], anti-breast cancer activities [12], etc. To date, many papers have reported the anti-breast cancer effects of traditional Chinese medicine formulas containing AM [13,14]. As the modernization of traditional Chinese medicine accelerates, increasing evidence suggests that AM is effective in the treatment of breast cancer with multi-targeted actions. Furthermore, the compounds from AM and their combinations with chemotherapeutic drugs have also been found to have promising anti-breast cancer effects. Here, we review the literature on this topic to comprehensively describe the pharmacological actions of AM against breast cancer, aiming to guide clinical practice and stimulate the exploration of innovative treatment medicines derived from AM in anti-breast cancer application. In this review, comprehensive literature searches were performed across the Web of Science (WOS) and PubMed databases. The search criteria included the term ‘Astragalus’ linked with ‘breast cancer’, along with individual searches for compounds from AM associated with ‘breast cancer’. Irrespective of publication dates, all references in line with the scope of this review have been included.

## 2. Breast Cancer Classification

Assessing the category of breast cancer is a prerequisite for its treatment, with the purpose of predicting tumor behavior, providing accurate disease diagnosis, and selecting the most appropriate treatment methods. Breast cancer is generally classified into non-invasive and invasive breast cancer based on histology. Non-invasive breast cancer typically opts for local treatment as the main therapeutic approach [15], while invasive breast cancer, due to its high metastatic potential, often requires systemic therapy. Based on the expression of estrogen receptors (ERs), progesterone receptors (PRs), human epidermal growth factor receptor 2 (HER2), and the measurement of tumor proliferation rate through the Ki67 level, breast cancer can be further classified into luminal A (ER- or PR-positive, or both; HER2-negative, low proliferation), luminal B (ER- or PR-positive, or both; HER2-negative, high proliferation), HER2-overexpressing, or triple-negative (ER, PR, and HER2-negative) [16]. Endocrine therapy is generally used for the treatment of luminal A breast cancer. Luminal B breast cancer, because of its high proliferative nature, is more sensitive to chemotherapy, and is typically treated with a combination of chemotherapy and endocrine therapy. HER2-overexpressing breast cancer is treated with chemotherapy plus targeted therapy, and endocrine therapy may also be added [17,18]. Triple-negative breast cancer (TNBC), which lacks multiple receptors, does not respond well to endocrine or targeted therapy. Therefore, surgery and chemotherapy may be the only viable treatment options for TNBC [19].

## 3. Chemical Compounds in AM

The research on AM chemical constituents is essential for understanding its pharmacological action. The root of AM is the principal part used in traditional medicine. The flavonoid content in the roots from different geographical origins can vary significantly. A study analyzed AM extracts from four different regions and identified 34 flavonoids, including 13 methoxylated flavones, 15 flavonoid glycosides, and 6 flavonol aglycones [20]. Another study measured the content of 14 major chemical constituents (5 flavonoids and 9 triterpenoid saponins) in 94 batches of AM from China, South Korea, and Germany [21]. These studies provide a foundation for the clinical application and further research of AM. Additionally, 31 compounds were identified in AM flowers from different sources, including 22 flavonoids, 8 isoflavonoids, and 1 benzopyran, with over 10 compounds showing significant differences in content among different sources, providing a basis for the pharmacological research of AM flowers [22]. Furthermore, a study determined the content of 11 isoflavonoids and 3 astragalosides in the xylem and bark of AM [23]. This research demonstrated that there are notable differences in the main active components in different parts of the AM stem. A summary of the components in different parts of AM can be found in Table S1 and Figure S1 of the Supplementary Materials.

## 4. Application of AM in the Treatment of Breast Cancer

As research on AM becomes increasingly in-depth, its chemical compounds, extracts, and formulas containing AM, and its use in conjunction with chemotherapeutic drugs have been discovered to have significant therapeutic effects on breast cancer.

### 4.1. Chemical Compounds

Compounds found in AM mainly include flavonoid compounds and saponin compounds. The anti-cancer effects of these compounds, including astragaloside IV [24], biochanin A [25], calycosin [26], formononetin [27], etc., have been reviewed. However, the anti-breast cancer effects are not the focus in these reviews. Here, to illustrate the substantial base of AM in anti-breast cancer, the effects and corresponding signal pathways of these compounds have been comprehensively reviewed in description, table, and figure forms. Table 1 summarizes the pharmacological effects of the main compounds in AM.

#### 4.1.1. Calycosin

Calycosin, a primary isoflavonoid compound extracted from AM, exhibits phytoestrogen characteristics, capable of binding to ER within the body and exerting estrogen-like effects [28]. Research has indicated that calycosin demonstrates significant potential in the field of breast cancer treatment [29], particularly in inhibiting the invasion and migration of breast cancer cells. Its mechanism of action includes the inhibition of basic leucine zipper transcription factor/transforming growth factor β1 (BATF/TGF-β1) signaling, thereby suppressing the epithelial–mesenchymal transition (EMT) of ER-positive breast cancer cells [29], and may also involve targeting the long non-coding RNA HOTAIR to suppress the progression of ER-positive/negative breast cancer cells [30]. Further studies have revealed that calycosin can reduce the migration and invasion capabilities of ER-positive breast cancer cells by targeting FoxP3-mediated vascular endothelial growth factor (VEGF) and matrix metalloproteinase (MMP)-9 expression [31]. However, whether calycosin operates through the same mechanisms in ER-negative breast cancer cell lines remains to be further investigated. Additionally, calycosin may inhibit the migration and invasion of TNBC cells by deactivating Rab27B-dependent signaling [32], offering a novel potential molecular mechanism for calycosin in the treatment of TNBC.

In ER-positive breast cancer cells, the proliferation inhibitory effect of calycosin is significant, which is associated with the inhibition of insulin-like growth factor 1 receptor (IGF-1R) induced by ERβ, the selective regulation of the mitogen-activated protein kinases (MAPK) and phosphoinositide 3-kinase/protein kinase B (PI3K/Akt) pathways [33], and the expression of miR-375 [34]. Although ER-negative breast cancer cell lines typically lack the expression of estrogen receptors, they may still express small amounts of ERβ, which could be the reason why calycosin also has an inhibitory effect on them [33]. Furthermore, calycosin may inhibit the growth of ER-negative breast cancer cells by stimulating the WDR7-7-GPR30 signaling pathway, downregulating SRC proto-oncogene (SRC) and epidermal growth factor receptor (EGFR), and inactivating the MAPK and PI3K/Akt pathways [35].

Calycosin has also been found to enhance mitochondrial apoptosis and promote the apoptosis of ER-positive breast cancer cells by stimulating the expression of the Ras-related dexamethasone-induced 1 (RASD1) gene [36,37]. Although these studies provide in vitro evidence, confirmation of in vivo efficacy still requires further research. Calycosin may also effectively inhibit the growth of ER-positive breast cancer by regulating the Akt signaling pathway and the expression of HOTAIR [38], representing the relationship between isoflavones and HOTAIR in breast cancer.

It is noteworthy that calycosin may inhibit apoptosis and promote the proliferation of ER-positive breast cancer cells at low concentrations [36,37,39,40]. At low concentrations (less than 16 μM), calycosin may inhibit apoptosis and promote the proliferation of ER-positive breast cancer cells (MCF-7) through the activation of ER and extracellular signal-regulated kinases 1 and 2 (ERK1/2) [39]. Furthermore, calycosin has been shown to stimulate the proliferation of endothelial cells (ECs) and breast cancer cells at low concentrations, but at a concentration of 20 μM, it stimulates the proliferation of ECs while inhibiting the growth of ERα-positive breast cancer cells (MCF-7 and T47D), possibly due to a feedback loop between ERα and RP11-65M17.3 [40].

Calycosin, AM water extract, astragaloside IV (AS-IV), and formononetin have all been discovered to activate the Nrf2-mediated signaling pathway, thereby inducing the expression of P-glycoprotein (P-gp) and breast cancer resistance protein (BCRP), and enhancing the efflux activity of these transporters [41]. This suggests that the concurrent use of these compounds with substrate drugs of P-gp and BCRP may lead to potential drug–drug interaction issues.

**Figure 1:**
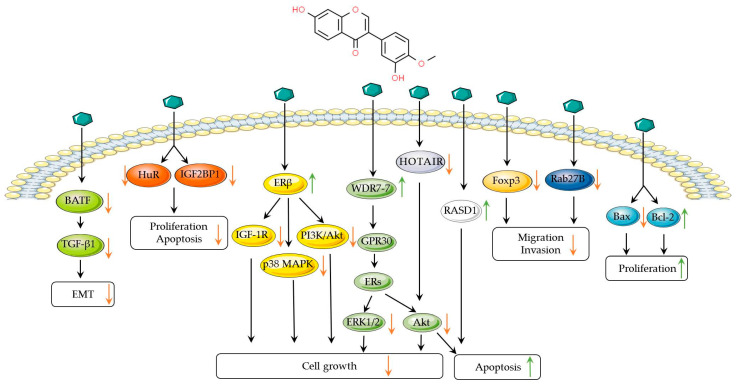
Signal pathways of calycosin in breast cancer. Calycosin can suppress EMT by inhibiting the BATF/TGF-β1 pathway. Calycosin targets the ER and downstream pathways to inhibit the proliferation of cancer cells. In addition, calycosin can promote apoptosis by inducing the Bax/BCL-2 pathway. Orange arrows indicate inhibitory effects, while green arrows represent inducing effects.

These findings (Figure 1) provide a scientific rationale for the potential application of calycosin in the treatment of various breast cancer, such as ER-positive breast cancer and TNBC, while also underscoring the necessity for further research to confirm its efficacy in vivo. Minor adverse effects or harmful responses may be observed after calycosin administration. The toxicity of calycosin has been reviewed recently [26], and will not be detailed here.

#### 4.1.2. Formononetin

Formononetin, an important phytoestrogen active component found in AM, has garnered widespread attention for its potential anti-cancer properties. Research indicates that it can effectively suppress the migration and invasion capabilities of TNBC cells by reducing the expression of MMP-2 and MMP-9 through the PI3K/AKT signaling pathway [42]. However, in-depth studies on whether formononetin can inhibit ER-positive cells through this pathway are lacking. Nonetheless, evidence suggests that formononetin may inhibit the proliferation and invasion of both ER-positive and ER-negative breast cancer cells by affecting the expression of programmed cell death 1 ligand 1 (PD-L1) and suppressing the activation of the stimulator of interferon genes—nuclear factor kappa B (STING-NF-κB) pathway [43]. Furthermore, through the BTB domain and CNC homology 1 (BACH1)/p53 signaling pathway, formononetin has the potential to reduce the invasiveness of TNBC and may improve the clinical prognosis of TNBC patients [44]. Although the sample size of relevant clinical studies is small, their findings still hold some reference value.

Further research has discovered that formononetin can inhibit the proliferation of ER-positive breast cancer cells by affecting the cell cycle progression through the suppression of the IGF1/IGF1R-PI3K/Akt signaling pathway [45]. Additionally, it has shown potential to improve drug resistance in TNBC, which may be associated with the regulation of the miR-199a-3p/mammalian target of rapamycin (mTOR) axis and the lncRNA AFAP1-AS1-miR-195/miR-545 axis [46,47], but these findings still require further investigation for validation. Formononetin can also inhibit the development of breast cancer by suppressing angiogenesis in ER-negative/positive breast cancer tumors, an effect achieved by targeting the fibroblast growth factor receptor 2 (FGFR2)-mediated Akt signaling pathway [48]. In ER-positive breast cancer, it also exerts this function by upregulating the expression of phosphatase and tensin homolog deleted on the chromosome ten (PTEN) protein and reducing the phosphorylation level of Akt [49]. It is noteworthy that formononetin has an inhibitory effect on the proliferation of ER-positive MCF-7 and T47D cells, but has no inhibitory effect on the growth of ER-negative breast cancer cells MDA-MB-435 S [50]. This indicates that the inhibitory effect of formononetin may vary among different cells, especially ER-negative cells, and further research is needed to clarify its different mechanisms of action in different types of breast cancer cells [50].

**Figure 2:**
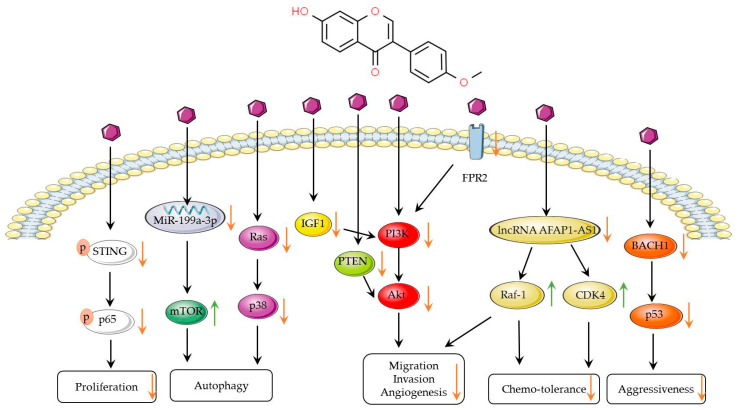
Signal pathways of formononetin in breast cancer. Formononetin can inhibit the proliferation, autophagy, invasion, and migration of breast cancer cells by suppressing the NF-κB p65, p38, Akt, and p53 signaling pathways. Formononetin can enhance autophagy through the MiR-199a-3p/mTOR pathway. Formononetin can modulate drug resistance by affecting the lncRNA AFAP1-AS1. Orange arrows indicate inhibitory effects, while green arrows represent inducing effects.

Beyond its potential application in breast cancer treatment (Figure 2), formononetin, due to its phytoestrogen characteristics, also provides a possible estrogen replacement therapy for postmenopausal women to alleviate a series of diseases caused by estrogen deficiency without increasing the risk of breast cancer [51]. Furthermore, formononetin is a promising safe treatment, and the toxicity of formononetin can be referenced in a recent review [27] (Figure 2).

#### 4.1.3. Astragaloside IV

Astragaloside IV, a low-toxic [24] triterpenoid saponin isolated from AM, has attracted attention among researchers for its broad range of biological activities, and its potential in the field of breast cancer treatment has been extensively investigated.

Firstly, astragaloside IV has been demonstrated to possess significant cytotoxicity and apoptosis-promoting effects on breast cancer cells. In one study, astragaloside IV, along with four other saponins (cyclocanthoside E, astrasieversianin X, macrophylosaponins B and D), was tested for its impact on ER-positive (MCF-7) and triple-negative (MDA-MB-231) breast cancer cells. The results indicated that these saponins could effectively reduce the viability of breast cancer cells and promote apoptosis [52]. Although this finding provides preliminary evidence for the anti-breast cancer potential of astragaloside IV, the specific mechanisms of action have not yet been fully elucidated.

Further research has unveiled the potential of astragaloside IV in inhibiting the proliferation and invasion of TNBC cells. Studies have found that astragaloside IV may achieve its inhibitory effects by downregulating Vav3 and suppressing the Ras-related c3 botulinum toxin substrate 1 (Rac1)/MAPK signaling pathway, as well as reducing the expression of MMP-2 and MMP-9 [12]. Additionally, astragaloside IV can disrupt the multidrug resistance and glycolysis of triple-negative doxorubicin-resistant breast cancer cell lines by blocking the hsa_circ_0001982-miR-206/miR-613 axis, thereby weakening their migration and invasion capabilities [53]. However, these studies have primarily focused on TNBC cells, and the mechanisms of action on other subtypes of breast cancer cells remain to be further explored.

**Figure 3:**
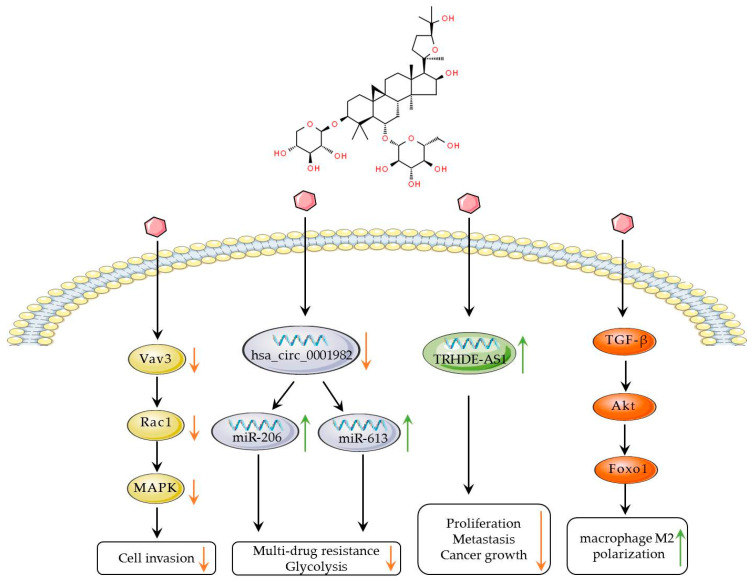
Signal pathways of astragaloside IV in breast cancer. Astragaloside IV can suppress cell invasion by inhibiting the MAPK signaling pathway and induce TRHDE-ASI to inhibit cell proliferation. Additionally, it can influence FOXO1, promoting the polarization of M2 macrophages. Orange arrows indicate inhibitory effects, while green arrows represent inducing effects.

It is noteworthy that lncRNA TRHDE-AS1 is closely related to breast cancer, and astragaloside IV can inhibit the proliferation and metastasis of breast cancer cells by upregulating the expression of TRHDE-AS1 both in vitro and in vivo [54]. This study may provide new diagnostic markers and therapeutic targets for the treatment of breast cancer. Furthermore, astragaloside IV has also been found to inhibit the polarization of M2-type macrophages by suppressing the TGF-β signal, thereby inactivating the Akt/forkhead box O1 (FoxO1) signaling pathway in macrophages, and subsequently reducing the proliferation, migration, and invasion capabilities of breast cancer cells [55]. This discovery provides further evidence of the multi-mechanism action of astragaloside IV in breast cancer treatment, indicating its potential for treating various types of breast cancer and leading the direction for future research (Figure 3).

#### 4.1.4. Biochanin A

**Figure 4:**
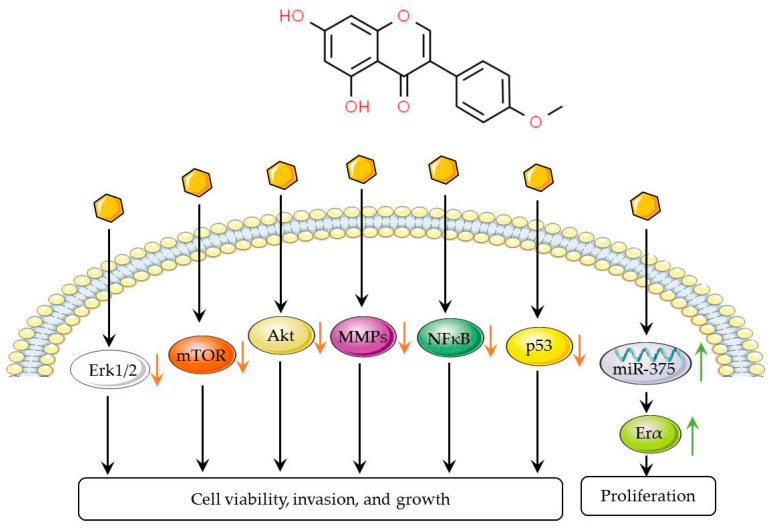
Signal pathways of biochanin A in breast cancer. Biochanin A can suppress the viability, invasion, and growth of breast cancer cells by inhibiting the Erk1/2, mTOR, Akt, NF-κB, and p53 signaling pathway, while it can enhance cell proliferation through the MiR-199a-3p/Era pathway. Orange arrows indicate inhibitory effects, while green arrows represent inducing effects.

Biochanin A, as an isoflavone identified from AM, has demonstrated activity against breast cancer [22] (Figure 4). Studies have shown that concentrations of biochanin A above 20 µg/mL can significantly suppress the growth of MCF-7 breast cancer cells, with this inhibitory effect being intricately linked to the expression of inducible nitric oxide synthase (iNOS) and the generation of nitric oxide [56]. Furthermore, biochanin A has been observed to markedly increase the activity of antioxidant and metabolic enzymes, which may contribute to the inhibition of cancer incidence in a prepubertal rat model [57], thus aiding in the reduction in the risk of breast tumor development. It is important to recognize that the study only confirms the ability of biochanin A to mitigate the risk of breast cancer induced through the same pathway as 7,12-dimethylbenzanthracene (DMBA).

At high concentrations (>50 μM), biochanin A influences the cell cycle of breast cancer cells by regulating the p53 protein, subsequently inhibiting cell growth, and it also curbs the proliferation of ER-positive breast cancer cells by suppressing DNA synthesis [58]. However, at low concentrations or levels akin to those of estrogen in healthy humans, biochanin A may paradoxically foster the proliferation of breast cancer cells [59,60]. Significantly, biochanin A exhibits a pronounced inhibitory effect on ER-negative/HER2-positive breast cancer cells, including the inhibition of cellular viability, signaling pathways, and the expression and activity of invasive enzymes, with no detrimental effect on the vitality of normal mammary cells and normal fibroblasts [61,62]. This discovery paves the way for new therapeutic approaches to ER-negative/HER-2-positive breast cancer. Furthermore, studies have shown the potential safety of biochanin A [25], which may support its further clinical use.

#### 4.1.5. Other Compounds

In addition to the aforementioned active components, several other bioactive compounds found in AM have been reported to exhibit anti-breast cancer activities. Astragaloside III has been demonstrated to possess anti-ER-positive breast cancer activity through both in vitro and in vivo experiments [63], although the specific mechanisms of its action have not yet been extensively investigated. Eight compounds isolated from AM have shown anti-proliferative activity against ER-positive breast cancer cells at a concentration of 45 μM [64]. TNBC is the most difficult subtype of breast cancer to cure. Diosmetin-7-O-rutinoside, a compound isolated from plants in the *Astragalus* genus in recent years, has shown effectiveness against both ER-positive and TNBC cell lines [65], although the mechanisms of its action remain unexplored. The active component ononin, found in AM, has been discovered to induce apoptosis in TNBC cells by triggering ferroptosis and disrupting the Nrf2/SLC7A11 axis [66], and researchers have also discussed its safety, indicating its promising potential for the treatment of TNBC. Furthermore, AM saponins I (astragaloside I), II (astragaloside II), and III (astragaloside III) have also shown good cytotoxicity and apoptosis-promoting effects on TNBC cells [67].

**Table 1:** The effects of chemical compounds from AM in breast cancer.

No.	Name	Ref.	Subjects	Dose	IC_50_ Value	Effects
1	Calycosin	[36]	MCF-7^(+++)^	0–200 μM	89.45 μM	RASD1↑, Bax/*BCL*-2↑, apoptosis↑
		[39]	MCF-7^(+++)^, ovariectomized mice	0–32 μM, 1–4 mg/kg/Day	33.6 μM, -	p-ERK1/2↑, apoptosis↓, proliferation↑
		[29]	T47D^(+++)^, MCF-7^(+++)^, T47D^(+++)^ xenograft tumor mouse model	0–400 μM, 0–400 μM, -	128.6 μM, 136.3 μM, -	BATF↓, TGFβ1↓, Ecadherin↑, N-cadherin↓, Vimentin↓, CD147↓, MMP-2↓, MMP-9↓, EMT↓, proliferation↓, invasion and migration↓
		[30]	MCF-7^(+++)^, T47D^(+++)^, MDA-MB-231^(---)^, SK-BR-3^(--+)^	0–80 μM for all models	56.1 μM, 58.4 μM, 75.6 μM, 92.5 μM	ERα-lncRNA HOTAIR↓, BRIP1↓ HuR↓, IGF2BP1↓ proliferation↓, apoptosis↑
		[40]	HUVECs, HMEC-1, MCF-7^(+++)^, T-47D^(+++)^, MCF-7^(+++)^ xenograft tumor mouse model	1–60 μM, 1–60 μM, 1–60 μM, 1–60 μM, 8 mg/kg/Day	-, -, 77.6 μM, 111.8 μM, -	RP11-65M17.3↓, ERK1/2↓, Akt phosphorylation↓, BRIP1↓, PARP-1↑ proliferation↑
		[31]	MCF-7^(+++)^, T47D^(+++)^	0–150 μM for all models	665.1 μM, 882.0 μM	Foxp3↓, VEGF↓, MMP-9↓, invasion and migration↓
		[32]	MDA-MB-231^(---)^	150 μM	-	Rab27B↓, β-catenin and VEGF↓ invasion and migration↓
		[33]	MDA-231^(---)^, MDA435^(---)^, MCF-7^(+++)^, T-47D^(+++)^	0–100 μM	311.6 μM, 395.3 μM, 47.7 μM, 61.6 μM	IGF-1R↓, ERβ↑, p38MAPK↑, Akt↓, PARP-1↓ proliferation↓, apoptosis↑
		[34]	MCF-7^(+++)^, T-47D^(+++)^, MDA-231^(---)^, MDA-435^(---)^	0–100 μM for all models	50.8 μM, 63.1 μM, 258.3 μM, 342.4 μM	Erβ↑, IGF-1R↓, PARP-1↑, miR-375↓, proliferation↓, apoptosis↑
		[38]	MCF-7^(+++)^	0–100 μM	52.0 μM	PI3K/Akt↓, HOTAIR↓, proliferation↓, apoptosis↑
		[35]	MDA-MB-468^(---)^, SK-BR-3^(--+)^, MDA-MB-231^(---)^, MCF-7^(+++)^, T-47D^(+++)^, MCF10A/MCF-7^(+++)^ xenograft tumor mouse model, SK-BR-3^(--+)^ xenograft tumor mouse model	1–32 μM, 1–32 μM, 1–32 μM, 1–32 μM, 1–32 μM, 1–32 μM, 55 mg/kg, 55 mg/kg	40.0 μM, 43.3 μM, -, 35.5 μM, 42.9 μM, 168.6 μM, -, -	p-SRC↓, p-EGFR↓, p-ERK1/2↓, p-Akt↓, R7-7↑, GPR30↓, proliferation↓
2	Formononetin	[42]	MDA-MB-231^(---)^, 4T1^(+++)^, MDA-MB-231^(---)^ xenograft tumor mouse model	0–160 μM, 0–160 μM, 10–20 mg/kg	638.1 μM, 2125.0 μM, -	PI3K/Akt↓, MMP-2↓, MMP-9↓, invasion and migration↓
		[43]	MCF-7^(+++)^, MDA-MB-468^(---)^	0–100μM for all models	151.9 μM, 163.5 μM	pSTING/STING↓, p-p65/p65↓, PD-L1↓, STING-NF-κB↓, proliferation↓, invasion↓
		[44]	MCF7^(+++)^, TNBC patients, MCF7^(+++)^ xenograft tumor mouse model	80 μM, -, -	-	BACH1↓, p53/PINK1/PARK2↑, BAX↑, caspase3↑, *BCL*↓, cytochrome C↑, caspases↑ apoptosis↑, invasion↓
		[46]	MDA-MB-231^(---)^, MDA-MB-231^(---)^/paclitaxel resistance, MDA-MB-231^(---)^/paclitaxel resistance xenograft tumor mouse model	0–80 μM, 0–80 μM, 30 mg/kg/3 day	1298.0 μM, 49.6 μM, -	miR-199a-3p↓, mTOR↑, drug resistance↓
		[45]	MCF-7^(+++)^, MCF-7^(+++)^ xenograft tumor mouse model	0–100 μM, 15–60 mg/kg/day	56.0 μM, -	IGF1/IGF1R-PI3K/Akt↓, p-IGF-1R↓, p-Akt↓, cyclin D1↓, proliferation↓
		[50]	MCF-7^(+++)^, T-47D^(+++)^, MDA-MB-435 S1^(---)^	0–100 μM for all models	51.3 μM, 65.9 μM, 177.6 μM	Ras-p38MAPK↑, p-p38↑, Bax/*BCL*-2↑, Ras↑, Raf-1↑, apoptosis↑
		[48]	HUVECs, T-47D^(+++)^, SK-BR-3^(--+)^, MCF-7^(+++)^ MDA-MB-231^(---)^, MDA-MB-231^(---)^ xenograft tumor mouse model	0–150 μM, 0–150 μM, 0–150 μM, 0–150 μM, 0–150 μM, 100 mg/kg/day	68 μM *, 31 μM *, 32 μM *, 27 μM *, 16 μM *, -	FGFR2 pathway↓, STAT3↓, PI3K↓, Akt↓, p-MMP-2/9↓, TGF-β↓, CD31↓, COX-2↓, angiopoietin-2↓, cell growth and angiogenesis↓
		[49]	MCF-7^(+++)^ xenograft tumor mouse model	0.1–0.4 mg/g	-	PTEN↑, p-Akt↓, VEGF↓, angiogenesis↓, apoptosis↑
		[47]	MDA-MB-231^(---)^, BT-549^(---)^, MCF-10A	0–160 μM for all models	200.9 μM, 349.4 μM, -	lncRNA AFAP1-AS1-miR-195/miR-545↓, proliferation↓, invasion and migration↓, drug resistance↓
		[34]	MCF-7^(+++)^, T-47D^(+++)^, MDA-231^(---)^, MDA-435^(---)^	0–100 μM for all models	75.0 μM, 75.3 μM, 211.2 μM, 342.4 μM	Erβ↑, IGF-1R↓, PARP-1↑, miR-375↓, proliferation↓, apoptosis↑
3	Astragaloside IV	[12]	MDA-MB-231^(---)^, MDA-MB-231^(---)^ xenograft tumor mouse model	0–100 μg/mL, 20 mg/kg/3 day	78.0 μg/mL, -	Vav3↓, Rac1/MAPK↓, ERK1/2↓, JNK↓, MMP-2↓, MMP-9↓, proliferation and migration↓
		[53]	MDA-MB-231/ADR^(---)^, BT-549/ADR^(---)^,MDA-MB-468/ADR^(---)^	40 μg/mL for all models	-	Hsa-circ-0001982-miR-206/miR-613↓, drug resistance↓, glycolysis↓, invasion and migration↓
		[54]	MCF-10A, MCF-7^(+++)^, MDA-MB-231^(---)^, MDA-MB-468^(---)^, MDA-MB-231^(---)^ xenograft tumor mouse model	0–80 μg/mL, 0–80 μg/mL, 0–80 μg/mL, 0–80 μg/mL, 20 mg/kg/3 day	-, 70.5 μg/mL, 89.9 μg/mL, -	TRHDE-AS1↑, MMP-2↓, MMP-9↓, proliferation↓, migration↓
		[55]	THP-1, MDA-MB-231^(---)^, MCF-7^(+++)^, MDA-MB-231^(---)^ xenograft tumor mouse model	0–100 μM, 0–100 μM, 0–100 μM, 0–100 μM, 40 mg/kg/day	-	TGF-β↓, Akt/FoxO1↓, TGF-β↓, M2 macrophage polarization↓, proliferation↓, invasion and migration↓
		[52]	MCF-7^(+++)^, MDA-MB-231^(---)^	0–200 μM for all models	32.41 μM	proliferation↓, apoptosis↑
4	Biochanin A	[60]	T47D^(+++)^, MCF-7^(+++)^	0–50 μM for all models	63.9 μM, 84.8 μM	miR-375↑, Erα↑, *BCL*-2↑, proliferation↑
		[61,62]	SK-BR-3^(--+)^, MCF-10A, NIH3T3	0–100 μM for all models	72.6 μM, 1749 μM, -	Erk1/2↓, Akt↓, mTOR↓, NF-κB↓, HER-2↓, MMP-9↓, MT-MMP1↓, cellular viability and invasive capacity↓
		[57]	SD rat	500 μg/g/2 day	-	SOD↑, catalase↑, GPx↑, GST↑, DT-diaphorase/GSH↑, LDH/LPO↓, antioxidant capacity↑
		[58]	T-47D^(+++)^	0–40 μg/mL	33.3 μg/mL	p53↑, cell division↓
5	Genistein	[38]	MCF-7^(+++)^	0–100 μM	59.8 μM	PI3K/Akt↓, HOTAIR↓, proliferation↓, apoptosis↑
6	Cyclocanthoside E, Astrasieversianin X, Macrophylosaponins B and D	[52]	MCF-7^(+++)^, MDA-MB-231^(---)^	0–200 μM for all models	cyclocanthoside E (54.74 μM *), astrasieversianin X (61.27 μM *), macrophylosaponins B (20.77 μM *), macrophylosaponins D (3.89 μM *)	proliferation↓, apoptosis↑
7	Astragaloside III	[63]	MCF-7^(+++)^, MCF-7^(+++)^ xenograft tumor mouse model	0–100 ng/mL, 0–20 mg/kg/2 day	10.2 ng/mL, -	apoptosis↑
8	Cycloartane glycosides	[64]	MCF-7^(+++)^	1–50 μM	-	cell viability↓
9	Diosmetin-7-O-rutinoside	[65]	MCF-10A, MCF-7^(+++)^, MDA-MB-231^(---)^	-	8.66 μg/m *, 13.65 μg/mL *, 12.89 μg/mL *	cellular necrosis↑
10	Astragaloside I, astragaloside II, astragaloside III	[67]	MDA-MB-231^(---)^	-	astragaloside I (0.209 mg/mL *), astragaloside II (0.205 mg/mL *), astragaloside III (0.194 mg/mL *)	cell viability↓, apoptosis↑
11	Ononin	[66]	MDA-MB-231^(---)^, 4 T1^(---)^, MDA-MB-231^(---)^ xenograft tumor mouse model	0–10 mM, 0–10 mM, 1–10 mg/kg/i.p	2.864 mM, 2.031 mM, -	Nrf2/SLC7A11↓, malondialdehyde↑, SOD↓, ROS↑, cell viability↓, apoptosis↑

“+++” indicates that the tests for ER, PR, and HER2 are all positive. “--+” indicates that the tests for ER and PR are negative, while the test for HER2 is positive. “---” indicates that the tests for ER, PR, and HER2 are all negative. “↑” and “↓” indicate activated and depressed, respectively. * These IC_50_ values are obtained from the corresponding reference. The other IC_50_ values are calculated with published data by using SPSS software v. 16.0 (SPSS Inc., Chicago, IL, USA), because of the IC_50_ value missing.

### 4.2. AM Extracts in the Treatment of Breast Cancer

As shown in Table 2, extracts of AM, including water extracts, alcohol extracts, AM polysaccharides, and total flavonoids, have gradually been recognized for their anti-breast cancer effects.

#### 4.2.1. AM Polysaccharides

AM polysaccharides, obtained through the process of water extraction, concentration, and purification of AM, have been confirmed to possess a variety of biological activities, including immune regulation, cardiovascular protection, antioxidant effects, and anti-cancer properties [68]. Particularly, AM polysaccharides have shown great potential in inhibiting TNBC. Studies have indicated that AM polysaccharides can suppress the proliferation, migration, and invasion of triple-positive and triple-negative breast cancer cells by inhibiting the Wnt/β-catenin signaling pathway and reducing EMT [69]. In vivo experiments with mice bearing triple-negative breast tumor models have shown a significant reduction in tumor volume, and this inhibitory effect may be related to the regulation of EGFR and annexin A1 (ANXA1) by AM polysaccharides [70]. Furthermore, AM polysaccharides can reduce the proliferation, migration, and invasion of triple-positive and triple-negative breast cancer cells by modulating cell cycle-related protein cyclin B1, cell division cycle 6 (CDC6), and the tumor suppressor protein p53 [71]. AM polysaccharides may also effectively inhibit the activity of TNBC cells by suppressing the PIK3CG/Akt/BCL2 signaling pathway, reducing invasiveness, and promoting apoptosis [72]. Additionally, AM polysaccharides may enhance the inhibitory effect of macrophages on EAC cancer cells by activating the Toll-like receptor 4 (TLR4)-mediated myd88-dependent signaling pathway [73].

Some studies have used in vivo circulating tumor cell capture methods to monitor the changes in the number of circulating tumor cells at different time points, proving that AM polysaccharides have an inhibitory effect on breast cancer metastasis [74]. Although this method can monitor and assess tumor metastasis, it can only detect circulating cancer cells in peripheral blood and cannot detect cancer cells that have entered the lymphatic circulation.

Interestingly, a study found that AM polysaccharides themselves have no inhibitory effect on the growth of TNBC cells but can activate RAW264.7 macrophages to release nitric oxide (NO) and tumor necrosis factor-α (TNF-α), thereby blocking the growth of breast cancer cells [75]. Subsequently, researchers conducted an in-depth exploration of the anti-tumor effects of AM polysaccharides and their potential mechanisms using immune cell cultures. They established an artificial lung scaffold tumor model with MCF-7 cells and assessed the inhibitory effect of the supernatant of RAW264.7 cells activated by AM polysaccharides on tumors [76]. The study found that this supernatant significantly inhibited the growth of three-dimensional (3D) cultured MCF-7 breast cancer cells, and the mechanism of action may involve inhibiting cell proliferation and inducing cell apoptosis. In addition, this anti-cancer cell activity may be related to the increased secretion of TNF-α and NO in RAW264.7 cells activated by AM polysaccharides. This study provides a new perspective for future research on anti-breast cancer drugs.

#### 4.2.2. Total Flavonoids from AM

The flavonoid components in AM, primarily isoflavones (potential ligands for ER), have not been extensively reported for the treatment of ER-positive breast cancer. There is only research indicating that total flavonoids from AM have an inhibitory effect on the proliferation of three TNBC cell lines and also exhibit an inhibitory effect on the migration of breast cancer cells [77]. Therefore, further in-depth research on the treatment of breast cancer with total flavonoids from AM is warranted.

#### 4.2.3. AM Water or Alcohol Extracts

AM water or alcohol extracts are active components containing a rich array of phenolic and flavonoid compounds with significant biological activities. Studies have shown that AM water or alcohol extracts have a significant preventive effect on DMBA-induced rat mammary tumors, which may be related to their antioxidant effects and the inhibition of oncogenic markers such as CA15.3, p53, and PCNA [78]. Additionally, AM extracts have been found to inhibit cell proliferation and induce apoptosis in triple-positive, triple-negative, and HER2-positive breast cancer cells through the PI3K/Akt/mTOR pathway [79]. Although these studies are primarily in vitro experiments, they provide a theoretical basis for the application of AM in the treatment of breast cancer. Interestingly, a three-dimensional fibrin gel was designed to simulate important characteristics of breast cancer in vivo, and the anti-proliferative effect of AM extracts on this model was demonstrated [80]. The design of this model offers a tool for the study of breast cancer treatment.

AM injection, a kind of AM extract, has also been found to inhibit the proliferation of TNBC cells and induce apoptosis. This effect may be achieved by downregulating the expression of EGFR and p53 proteins [81,82]. It is important to note that AM compounds may have estrogen-like effects at low concentrations, which could explain their promotional effects in certain situations.

Furthermore, in clinical studies, AM tincture has been found to maintain peripheral blood white blood cells and absolute neutrophils in breast cancer chemotherapy patients [83], suggesting that AM tincture may help to counteract the immunosuppressive side effects of chemotherapy.

**Table 2:** The effects of extracts from AM in breast cancer.

No.	Name	Ref.	Subjects	Dose	IC_50_ Value	Effects
1	AM polysaccharides	[69]	MCF-7^(+++)^, MDA-MB-231^(---)^	800 μg/mL for all models	836.7 μg/mL, 668.1 μg/mL	Wnt/β-catenin↓, snail↓, vimentin↓, E-cadherin↑, proliferation↓, invasion and migration↓
		[84]	MCF-7^(+++)^, RAW264.7	0–1 mg/mL for all models	-	NO↑, TNF-α↑, Bax/BCL-2↑, apoptosis↑
		[70]	4T-1^(---)^ xenograft tumor mouse model	0–200 mg/kg/day	-	EGFR↓, ANXA1↑, apoptosis↑, cell growth↓
		[71]	MCF-7^(+++)^, MDA-MB-231^(---)^	0–2 mg/mL for all models	2.089 mg/mL, 0.823 mg/mL	Cyclin B1↓, CDC6↓, p53↑, proliferation↓, invasion and migration↓
		[74]	4T-1^(---)^ xenograft tumor mouse model	300 mg/kg/day	-	migration↓
		[73]	RAW 264.7, TLR4 deficient mice, myd88 deficient mice, EAC xenograft tumor mouse model	400 μg/mL, 500 mg/kg/day, 500 mg/kg/day, 500 mg/kg/day	-	TLR4-MyD88↑, NO↑, IL-1β↑, IL-6↑, TNF-α↑, cellular immune response↑
		[72]	MDA-MB-231^(---)^	0–2 mg/mL	1.08 mg/mL	PIK3CG/Akt/BCL2↓, p-Akt↓, cell viability↓, invasion↓
		[75]	RAW264.7, 4T-1^(---)^, 4T-1^(---)^ xenograft tumor mouse model	0–1000 μg/mL, 0–1000 μg/mL, 100–200 mg/kg/day	-	TNF-α↑, IFN-γ↑, Bax/BCL-2↑, caspase-9↑, caspase-3↑, cellular immune response↑
2	The supernatant liquid of AM polysaccharide-treated RAW264.7 cell	[76]	RAW264.7, MCF-7^(+++)^ tissue-engineered tumor model	0, 500, 1000 μg/mL (AM polysaccharide)	-	TNF-α↑, NO↑, cellular immune response↑, BCL-2↓, Bax↑, apoptosis↑
3	AM flavonoids	[77]	MDA-MB-468^(---)^, MDA-MB-361^(---)^, BT20^(---)^, MDA-MB-468^(---)^, MDAMB-361^(---)^, BT20^(---)^ xenograft tumor mouse model	0–2 mg/mL, 0–2 mg/mL, 0–2 mg/mL, 0–400 mg/kg, 0–400 mg/kg, 0–400 mg/kg	1.300 mg/mL, 2.847 mg/mL, 2.982 mg/mL, -, -, -	BCL-2↑, caspase-9↑, BRCA1↑, FAK↓, apoptosis↑, invasion and migration↓
4	Water extract of AM	[78]	DMBA-induced rat breast cancer model	0–240 mg/kg/day	-	CA15.3↓, p53↓, MDA↓, calcium ions↓, PCNA↓, CAT↑, exert prophylactic effect, reduce the pathological deformity of breast cancer tissue
		[79]	MCF-7^(+++)^, SK-BR-3^(--+)^, MDA-MB-231^(---)^	0–100 μg/mL for all models	61.1 μg/mL, 86.8 μg/mL, 58.7 μg/mL	p-PI3K↓, p-GSK3β↓, p-Akt↓, p-mTOR↓, total-mtor↑, proliferation↓, apoptosis↑
		[85]	MDA-231^(---)^, AGS, KATO-III, HT29, Mel7, Mel14	0–100 μg/mL for all models	-, 34.9 μg/mL, -, -, -, -	cell growth↓
5	70% ethanol extract of AM	[80]	MCF-7^(+++)^ scaffold-based 3D model	0–2000 μg/mL	253.2 μg/mL *	caspase-3↑, caspase-8↑, caspase-9↑, Ki-67↓, proliferation↓, apoptosis↑
6	AM injection	[81]	MDA-MB-231^(---)^	0–200 mg/mL	3.5 mg/mL	proliferation↓, apoptosis↑
		[82]	MDA-MB-468^(---)^	0–1 g/mL	-	EGFR↓, p53↓, proliferation↓, apoptosis↑
7	AM tincture	[83]	breast cancer patients	10 mL/day	-	maintaining the numbers of peripheral blood white blood cells and absolute neutrophils in breast cancer patients after chemotherapy

“+++” indicates that the tests for ER, PR, and HER2 are all positive. “--+” indicates that the tests for ER and PR are negative, while the test for HER2 is positive. “---” indicates that the tests for ER, PR, and HER2 are all negative. “↑” and “↓” indicate activated and depressed, respectively. * This IC_50_ value is obtained from the corresponding reference. The other IC_50_ values are calculated with published data by using SPSS software (SPSS Inc., Chicago, IL, USA), because of the IC_50_ value missing.

### 4.3. Traditional Chinese Medicine Formulas Containing AM

A herbal extract known as SH003, which is composed of AM, *Angelica sinensis* (Dang Gui), and *Trichosanthes kirilowii* (Gua Lou), has been demonstrated to inhibit the growth and metastasis of TNBC cells by activating the p73 pathway [86] or suppressing the activity of STAT3 [87]. Moreover, this formula has been shown to ameliorate adverse reactions that may occur when Dang Gui and Gua Lou are used individually [87]. Further research has found that this formula can induce autophagy by inhibiting STAT3 and mTOR, and by mediating the production of reactive oxygen species (ROS) through lysosomal p62/SQSTM1 accumulation, thereby slowing tumor growth [88]. Additionally, this formula has been discovered to inhibit drug resistance in paclitaxel-resistant TNBC cells by suppressing the STAT3 signaling pathway [89]. Studies also indicate that a herbal formula (KSG-002) extracted from AM and Dang Gui with a 3:1 ratio (*w*/*w*) can inhibit the growth and metastasis of TNBC by targeting NF-κB-mediated TNF-α production in macrophages [90]. However, the optimal combination ratio of these two medicinal materials has not yet been determined, and further research is needed to optimize it. Furthermore, RLT-03 extracted from AM, *Lonicerae japonicae* Flos (Jin Yin Hua), *Trichosanthes kirilowii* (Tian Hua Fen), and *Imperata cylindrica* (Bai Mao Gen) have shown potential therapeutic effects on TNBC by downregulating the expression of receptor tyrosine kinase (RTK) ligands and inflammatory factors [13]. Interestingly, extracts of medicinal mushrooms (*Coriolus versicolor*, *Ganoderma lucidum*, *Phellinus linteus*), medicinal herbs (*Scutellaria barbata*, AM, *Curcuma longa*), and purified biologically active nutritional compounds (diindolylmethane and quercetin) have been found to significantly inhibit the proliferation and metastatic behavior of aggressive triple-negative human breast cancer cells, with a stronger effect on inhibiting metastasis than proliferation, and without observed drug toxicity [14,91]. Lastly, the San Huang Tang formula (composed of AM, *Rheum palmatum*, and *Curcuma longa*) has been shown to modulate inflammation and oxidative stress responses through the upregulation of nuclear factor erythroid 2-related factor 2 (Nrf2) via the PI3K/AKT/mTOR signaling pathway in a triple-positive breast cancer cell xenograft nude mouse model, and can significantly promote severe necrosis and induce cell death [92]. Fangji Huangqi Tang, which includes *Stephania tetrandra*, AM, *Glycyrrhiza uralensis*, and *Atractylodes macrocephala*, originates from the “Synopsis of Golden Chamber” by Zhang Zhongjing. Modern research has proven that it can inhibit the proliferation of MB-MDA-231 cells and induce apoptosis after a 24 h treatment. Unfortunately, there is currently a lack of research on the underlying mechanisms of its actions [93]. The results of these studies are summarized in Table 3.

**Table 3:** The effects of traditional Chinese medicine formulas containing AM in breast cancer.

No.	Prescription	Ref.	Subjects	Dose	IC50 Value	Effects
1	SH003 (containing AM, *Angelica sinensis*, and *Trichosanthes kirilowii* with 1:1:1 ratio)	[86]	Hs578T^(---)^, MDA-MB-231^(---)^, ZR-751^(++-)^, MCF-7^(+++)^, T-47D^(+++)^	0–200 μg/mL for all models	124.4 μg/mL, 115.0 μg/mL, 165.4 μg/mL, 477.3 μg/mL, -	PARP cleavage protein↑, p73↑, apoptosis↑
		[87]	MCF-7^(+++)^, T-47D^(+++)^, SK-BR-3^(--+)^, BT-20^(---)^, MDA-MB-231^(---)^, GBL-60/MDA-MB-231^(---)^ xenograft tumor mouse model	0–500 μg/mL, 0–500 μg/mL, 0–500 μg/mL, 0–500 μg/mL, 0–500 μg/mL, 500 mg/kg/day	63.5 μg/mL, 405.2 μg/mL, 532.8 μg/mL, -, 105.4 μg/mL, 201.6 μg/mL, -	STAT3-IL-6↓, PARP cleavages↑, p-STAT3↓, IL-6↓, cyclin D↓, MMP-9↓, VEGF↓, survivin↓, cell growth↓, invasion and migration↓
		[88]	MDA-MB-231^(---)^, HCC-38^(---)^/MDA-MB-231^(---)^ xenograft tumor mouse model	0–500 μg/mL, 0–500 μg/mL, 500 mg/kg/day	47.2 μg/mL, 144.7 μg/mL,-	cathepins↓, STAT3-mtor↓, p62↑, p62/SQSTM1↑, ROS↑, cell growth↓, apoptosis↑
		[89]	MCF-7^(+++)^, MCF-7^(+++)^/paclitaxel-resistant	0–1000 μg/mL,	47.2 μg/mL, 144.7 μg/mL	STAT3↓, P-gp↓, MRPs↓, VEGF↓, MMP-2↓, drug resistance↓
2	KSG-002 (containing AM and *Angelica sinensis* with 3:1 ratio)	[90]	MCF-10A, MDA-MB-231^(---)^, BT-20^(---)^, Raw264.7/MDA-MB-231^(---)^ xenograft tumor mouse model,	0–500 μg/mL, 0–500 μg/mL, 0–500 μg/mL, 0–500 μg/mL, 500 mg/kg/day	-	NF-*κ*B↓, TNF-α↓, iNOS↓, COX-2↓, MMP-9↓, Fas↓, proliferation and migration↓
3	RLT-03 (containing AM, *Lonicerae japonicae Flos*, *Trichosanthes kirilowii*, and *Imperata cylindrica*)	[13]	4T-1^(---)^, EMT-6^(---)^, BT-549^(---)^, MDA-MB-231^(---)^, EMT6^(---)^ xenograft tumor mouse model	0–7.5 mg/mL, 0–7.5 mg/mL, 0–7.5 mg/mL, 0–7.5 mg/mL, 20mg/g/day	2.387 mg/mL *, 2.002 mg/mL *, 2.583 mg/mL *, 0.638 mg/mL *, -	RTK ligands↓, VEGF↓, EGF↓, IL-10↓, TGF-β↓, CD34↓, proliferation↓, apoptosis↑
4	Sanhuang decoction (containing AM, *Rheum palmatum*, and *Curcuma longa* with 3:1:1 ratio)	[92]	MCF-7^(+++)^ xenograft tumor mouse model	6.4 g/kg/2 day	-	PI3K/Akt/mTOR↓, Nrf2↑, TNF-α↑, IL-6↓, SOD↓, TAOC↓, MDA↓, VEGF↓, MMP-2↓, MMP-9↓, apoptosis↑, regulating inflammation and oxidative stress
5	BreastDefend™ (containing *Coriolus versicolor*, *Ganoderma lucidum*, *Phellinus linteus*, *Scutellaria barbata*, AM, *Curcuma longa*, diindolylmethane, and quercetin)	[14]	MDA-MB-231^(---)^ xenograft tumor mouse model	0–400 mg/kg	-	PLAU↓, CXCR4↓, migration↓
6	AM and *Vaccaria hispanic* with 1:1 ratio	[94]	MCF7^(+++)^, A549, T24, PANC-1, U-2 OS	0–2 mg/mL for all models	0.5 mg/mL, 0.7 mg/mL, 1.0 mg/mL, 0.7 mg/mL, 0.2 mg/mL	Akt↑, ERK1/2↑, p21↓, p27↓, apoptosis↑
7	Fangji Huangqi decoction (containing *Stephania tetrandra*, AM, *Glycyrrhiza uralensis*, *Atractylodes macrocephala* with 4:5:2:3 ratio)	[93]	MB-MDA-231	0–64 mg/mL	5.28 mg/mL *	apoptosis↑

“+++” indicates that the tests for ER, PR, and HER2 are all positive. “--+” indicates that the tests for ER and PR are negative, while the test for HER2 is positive. “---” indicates that the tests for ER, PR, and HER2 are all negative. “++-” indicates that the tests for ER and PR are positive, while the test for HER2 is negative. “↑” and “↓” indicate activated and depressed, respectively. * This IC_50_ value is obtained from the corresponding reference. The other IC_50_ values are calculated with published data by using SPSS software (SPSS Inc., Chicago, IL, USA), because of the IC_50_ value missing.

### 4.4. Combination Therapy Involving AM

The integration of AM with chemotherapeutic agents expands the horizons of chemotherapy, presenting a promising research avenue. Research has indicated that the synergistic application of calycosin and everolimus significantly suppresses the proliferation of TNBC cells and enhances apoptosis, outperforming the effects of single-agent therapy [95]. However, the safety assessment of this combination was primarily based on body weight changes in animal models, which may have certain limitations. The combined use of formononetin and metformin has also demonstrated an inhibitory effect on the growth of triple-positive breast cancer cells, inducing apoptosis [96], yet further validation through in vivo experiments is warranted. Astragaloside IV, derived from AM, enhances the chemosensitivity of breast cancer to paclitaxel by inhibiting caveolin-1 (CAV-1) and activating the endothelial nitric oxide synthase/nitric oxide/peroxynitrite (eNOS/NO/ONOO^−^) signaling pathway, highlighting the benefits of its combination with paclitaxel in breast cancer treatment [97]. It is also valuable to explore whether AM could similarly potentiate the effects of other chemotherapeutic drugs. Moreover, the concomitant use of AM polysaccharides, α-solanine, lotus alkaloid, and 2,3,5,6-tetramethylpyrazine has been shown to significantly curb the proliferation and migration of TNBC cells, while also enhancing autophagy flux [98].

Stemness of cancer cells is a primary cause of chemotherapy failure and recurrence in breast cancer. Astragaloside IV has been found to diminish the stemness of triple-positive breast cancer stem cells, augmenting the sensitivity to chemotherapy and synergistically promoting apoptosis in breast cancer cells induced by paclitaxel [99]. Concurrently, the combination of AM and *Vaccharia hispanica* exhibits synergistic anti-proliferative and cytotoxic effects on cancer cells, triggering over-activation of the Akt and ERK1/2 pathways, downregulating p21 and p27, and disrupting the cancer cell cycle, leading to accumulation at the G2/M phase and subsequent apoptosis [94]. However, this study was specific to the MCF7 cell line, and the potential impact on TNBC warrants further investigation.

The combined therapy of Shenqi Fuzheng injection with cisplatin has been shown to enhance the sensitivity of MDA-MB-231/DDP+M2 cells to cisplatin and reverse cisplatin resistance mediated by M2 macrophages via the PI3K pathway [100]. Additionally, the co-administration of oxymatrine and AM polysaccharides may bolster the immune system’s capacity to combat TNBC by improving the infiltration and anti-tumor functionality of tumor-infiltrating lymphocytes [101]. The synergistic use of biochanin A, an active component of AM, and ginsenoside Rh2 has also exhibited anti-proliferative effects on triple-negative and triple-positive breast cancer cells, intensifying the inhibition of breast cancer cell migration and invasion [102].

Doxorubicin’s cardiotoxicity is a significant constraint on its therapeutic application. However, the combined use of astragaloside IV and doxorubicin can mitigate doxorubicin-induced apoptosis in cardiomyocytes without compromising its anti-tumor efficacy against breast cancer cells [103], thereby broadening the therapeutic window for doxorubicin. This conclusion, however, necessitates further clinical data for validation. AM polysaccharides have been shown to reverse the paclitaxel-induced alterations in the cell cycle and apoptosis of macrophages RAW 264.7, and their combination with paclitaxel demonstrates a synergistic effect on survival rates without potentially undermining the chemotherapeutic impact on cancer cells [104]. This approach could significantly reduce the immunosuppressive effects of paclitaxel on the immune system while maintaining effective treatment. AM polysaccharides, when combined with 5-fluorouracil (5-FU), can also enhance anti-tumor outcomes and mitigate the immunosuppressive effects of 5-FU on the immune system [75]. The combined use of a mixed extract of AM, *Angelica sinensis*, and *Trichosanthes kirilowii* with doxorubicin has been shown to improve treatment outcomes for TNBC with minimal or no side effects [105], and it has also been discovered that this mixed extract, when used in conjunction with paclitaxel, can overcome paclitaxel resistance in breast cancer cells [106], offering promising clinical potential. The results of the aforementioned studies are presented in Table 4.

**Table 4:** The effects of AM and its compounds in the combination therapy of breast cancer.

No.	Combination (Dose)	Subjects	Effects	Ref.
1	Formononetin (0–150 μM, 50 mg/kg/day) Everolimus (0–2000 nM, 2 mg/kg/day)	MDA-MB-468^(---)^/MDA-MB-468^(---)^ xenograft tumor mouse model	mTORC2↓, p-mTOR↓, p-p70S6K↓, PTEN↑, p-4EBP-1↑, proliferation↓, apoptosis↑, enhancing the effect of everolimus in inhibiting the growth of breast cancer cells	[95]
2	Astragaloside IV (0–90 μM, 0–90 μM, 50 mg/kg/day) Paclitaxel (0–70 nM, 0–70 nM, 10 mg/kg/day)	MCF-7^(+++)^, MDA-MB-231^(---)^, MDA-MB-231^(---)^ xenograft tumor mouse model	CAV-1↓, eNOS/NO/ONOO−↑, p-ERK1/2↓, p-JNK↓, p-p38↑, enhancing paclitaxel sensitivity in breast cancer cells	[97]
3	Astragaloside IV (0–60 nM, 0–60 nM, 40 mg/kg/2 day) Paclitaxel (0–1 μM, 0–1 μM, 5 mg/kg/2 day)	MCF7^(+++)^ with stemness, xenograft tumor mouse model using MCF-7^(+++)^ with/without stemness	Sox2/Nanog↓, breast cancer plasticity↓, drug resistance↓	[99]
4	Astragaloside IV (0–60 μM, 15 mg/kg/day) α-Solanine (0–64 μM, 5 mg/kg/day) Neferine (0–80 μM, 10 mg/kg/day) 2,3,5,6-tetramethylpyrazine (0–750 μM, 5 mg/kg/day)	MDA-MB-231^(---)^/MDA-MB-231^(---)^ xenograft tumor mouse model	ATG16L1↑, ATG9B↑, ATG4D↑, TMEM74↓, TNF↓, HB-EGF↓, thrombospondin-2↓, amphiregulin↓, leptin↓, IGFBP-9↓, EGF↓, coagulation factor III↓, MMP-9↓, serpin E1↑, PF4↑, proliferation and migration↓, autophagy↑	[98]
5	AM polysaccharides (0–100 μg/mL, 40 mg/kg/day) Paclitaxel (10 μM, 20 mg/kg/week)	4T1^(---)^, 4T1^(---)^ xenograft tumor mouse model, RAW 264.7	P-H2A↓, PARP↓, Chk1↓, p53↓, p21↓, BCL-Xl↑, MCL-1↑, the toxicity of paclitaxel to normal cells↓	[104]
6	AM polysaccharides (200 mg/kg) 5-FU (20 mg/kg)	4T-1^(---)^ xenograft tumor mouse model	The antitumor effect of 5-FU↑, the immunosuppressive effect of 5-FU on the immune system↓	[75]
7	Shenqi Fuzheng injection (0–320 μL/mL, 0–60 mg/kg/day) Cisplatin, (0–120 μM, 2 mg/kg/day)	MDA-MB-231/DDP^(---)^ + M2,/MDA-MB-231/DDP^(---)^ + M2 xenograft tumor mouse model	PI3K pathway↑, P-gp↓, ABCG2↓, CD206↓, CD86↑, PGE2, IL-6, CCL1↓, BCL-2↓, Bax↑, drug resistance↓, anti-tumor effect↑	[100]
8	Astragaloside IV (15–30 μg/mL, 15–30 μg/mL) Doxorubicin (1 μM, 1 μM)	MCF-7^(+++)^, neonatal rat cardiomyocytes	PI3K/Akt↑, ROS↓, LDH↓, CK-MB↓, cytochrome C↓, doxorubicin-induced decrease in ATP↓, SDH↑, ATP synthase activity↑, doxorubicin-induced mitochondrial damage and dysfunction↓, the cardiotoxicity of doxorubicin on cardiomyocytes↓	[103]
9	Extract from AM, *Angelica sinensis*, and *Trichosanthes kirilowii* (0–500 μg/mL, -) Doxorubicin (0–1000 nM, -)	MDA-MB-231^(---)^, MDA-MB-231^(---)^ xenograft tumor mouse model	Bax↑, Caspases↑, PARP cleavages↑, BCL-2↓	[105]
10	Extract from AM, *Angelica sinensis*, and *Trichosanthes kirilowii* (0–500 μg/mL, 0–500 μg/mL) Paclitaxel (0–1000 nM, 0–1000 nM) Tamoxifen (0–20 μM, 0–20 μM)	MCF-7^(+++)^, paclitaxel-resistant MCF-7^(+++)^	P-gp↓, drug resistance↓	[106]
11	Formononetin (0–160 μM) Metformin (0–300 μM)	MCF-7^(+++)^	ERK1/2↓, BCL-2↓, p-ERK1/2↓, proliferation↓	[96]
12	Astragaloside IV (0–8 μg/mL, 0–8 μg/mL, 0.6–0.8mg/kg/day) Oxymatrine (0–4 μg/mL, 0–4 μg/mL, 1.2–1.6 mg/kg/day)	CTLL-2, 4T-1^(---)^, NIH3T3/4T-1^(---)^ xenograft tumor mouse model	FAP↓, α-SMA↓, CAF activation↓, T cells infiltrate through CAFs↑, the antitumor effect of immune cells↑	[101]
13	Biochanin A (30–70 μM, 30–70 μM) Ginsenoside Rh2 (30–70 μM, 30–70 μM)	MDA-MB-231^(---)^, MCF-7^(+++)^	p-p53↑, p-p38↑, p-ASK1↑, TRAF2↓ proliferation↓, enhancing the inhibition of cell invasion and migration↑	[102]
14	Biochanin A (5, 15 mg/kg/day) Quercetin (5 mg/kg/day) Epigallocatechin-3-gallate (5 mg/kg/day)	MCF-7^(+++)^ xenograft tumor mouse model	Tumor size↓	[107]

“+++” indicates that the tests for ER, PR, and HER2 are all positive. “---” indicates that the tests for ER, PR, and HER2 are all negative. “↑” and “↓” indicate activated and depressed, respectively.

## 5. Conclusions

The therapeutic role of AM in breast cancer is becoming more defined, exhibiting a multifaceted mechanism that targets multiple pathways and molecular targets. Compounds isolated from AM, including astragaloside IV, biochanin A, calycosin, and formononetin, have been demonstrated to exert promising anti-breast cancer activities. Notably, phytoestrogens such as calycosin and formononetin have been observed to stimulate breast cancer cell proliferation at low concentrations, akin to those found in human estrogen levels. This underscores the complexity of their interactions with breast cancer cells. In comparison to standard breast cancer therapies, AM extracts, traditional Chinese medicine formulations, and prepared Chinese medicinal products have shown promise in research for their therapeutic and adjuvant benefits in breast cancer treatment. Their advantages lie in enhancing and restoring immune functions, overcoming drug resistance, and directly inhibiting cancer cell proliferation and inducing apoptosis. The synergistic use of AM with other therapeutic agents has the potential to reduce side effects and enhance treatment efficacy, thereby broadening their therapeutic horizon.

The diverse receptor profiles of different breast cancer types suggest varying mechanisms of action for AM in combating the disease. To optimize the use of AM in breast cancer therapy, a comprehensive investigation of its mechanisms of action is essential. On the other hand, current methodologies for assessing the efficacy of breast cancer treatments primarily involve in vitro experimentation with various breast cancer cell lines and in vivo studies using mouse tumor xenograft models. However, these approaches do not fully replicate the complex tissue microenvironment of breast cancer. Hence, the development of more representative models for drug efficacy evaluation represents a significant avenue for future investigative efforts. Furthermore, nanocarrier delivery systems hold significant potential for the treatment of cancer. This could be an effective avenue for enhancing the efficacy of AM-derived medicines and represents one of the promising directions for future development.

## Supplementary Materials

The following supporting information can be downloaded at: https://www.mdpi.com/article/10.3390/biom14101339/s1, Figure S1: Chemical structure of substance in *Astragalus membranaceus*; Table S1: Substances found in *Astragalus membranaceus*.

## Abbreviations

AM, *Astragalus membranaceus*; ER, estrogen receptor; PR, progesterone receptor; HER2, human epidermal growth factor receptor 2; TNBC, triple-negative breast cancer, BATF/TGF-β1, basic leucine zipper transcription factor/transforming growth factor β1; EMT, epithelial–mesenchymal transition; VEGF, vascular endothelial growth factor; MMP, matrix metalloproteinase; IGF-1R, insulin-like growth factor 1 receptor; MAPK, mitogen-activated protein kinases; PI3K/Akt, phosphoinositide 3-kinase/protein kinase B; SRC, SRC proto-oncogene; EGFR, epidermal growth factor receptor; RASD1, Ras-related dexamethasone-induced 1; ERK1/2, extracellular signal-regulated kinases 1 and 2; ECs, endothelial cells; P-gp, P-glycoprotein; BCRP, breast cancer resistance protein; HuR, human antigen R; IGF2BP1, insulin-like growth factor 2 mRNA-binding protein 1; BAX, BCL-2-associated X protein; BCL-2, B-cell lymphoma 2; PD-L1, programmed cell death 1 ligand 1; STING-NF-κB, stimulator of interferon genes-nuclear factor kappa B; BACH1, BTB domain and CNC homology 1; mTOR, mammalian target of rapamycin; FGFR2, fibroblast growth factor receptor 2; PTEN, phosphatase and tensin homolog deleted on chromosome ten; FPR2, formyl peptide receptor 2; Raf-1, Ras proto-oncogene serine/threonine-protein kinase; CKD4, chronic kidney disease stage 4; CDK4, cyclin-dependent kinase 4; Rac1, Ras-related c3 botulinum toxin substrate 1; iNOS, inducible nitric oxide synthase; DMBA, 7,12-dimethylbenz-anthracene; BRIP1, BRCA1-interacting helicase 1; PARP-1, poly(ADP-ribose) polymerase 1; GPR30, G protein-coupled receptor 30; PINK1/PARK2, PTEN-induced putative kinase 1/Parkin RBR E3 ubiquitin protein ligase; STAT3, signal transducer and activator of transcription 3; FoxO1, forkhead box O1; GPx, glutathione peroxidase; SOD, superoxide dismutase; GST, glutathione S-transferases; LDH/LPO, lactate dehydrogenase/lipid peroxidation; ROS, reactive oxygen species; ANXA1, annexin A1; TLR4, Toll-like receptor 4; TNF-α, tumor necrosis factor-α; CDC6, cell division cycle 6; MyD88, myeloid differentiation primary response protein 88; IL-1β, interleukin-1β; FAK, focal adhesion kinase; PCNA, proliferating cell nuclear antigen; GSK-3β, glycogen synthase kinase-3 beta; ROS, reactive oxygen species; RTK, receptor tyrosine kinase; Nrf2, nuclear factor erythroid 2-related factor 2; MRP, multiple resistance protein; Fas, factor-related apoptosis; TAOC, total antioxidant capacity; MDA, malondialdehyde; PLAU, urokinase plasminogen activator; CXCR4, C-X-C motif chemokine receptor 4; CAV-1, caveolin-1; 4EBP-1, eukaryotic translation initiation factor 4E-binding protein 1; JNK, c-Jun N-terminal kinase; ATG, autophagy-related; TMEM, transmembrane protein; HB-EGF, heparin-binding epidermal growth factor-like growth factor; IGFBP, insulin-like growth factor binding protein; PF4, platelet factor 4; P-H2A, phosphorylated histone H2A; Chk1, checkpoint kinase 1; MCL-1, myeloid cell leukemia 1; ABCG2, ATP-binding cassette subfamily G member 2; PGE2, prostaglandin E2; CCL1, chemokine (C-C motif) ligand 1; Bax, BCL-2-associated X protein; CK-MB, creatine kinase isoenzymes; FAP, fibroblast activation protein; α-SMA, α smooth muscle actin; CAFs, cancer-associated fibroblasts; TRAF2, tumor necrosis factor receptor-associated factor 2.
