# Supplementary material for: Astragalus membranaceus: A Traditional Chinese Medicine with Multifaceted Impacts on Breast Cancer Treatment
Source: Biomolecules. 2024 Oct 21;14(10):1339. doi: 10.3390/biom14101339 (PMC11506204; doi:10.3390/biom14101339)
Supplement: Supplementary file 1 [file biomolecules-14-01339-s001.zip › biomolecules-3240716-supplementary.pdf]

Table S1. Substances found in *Astragalus membranaceus*.

No.	Source	Name	Elemental composition	CAS	Ref.
1	Flower, root	Liquiritin	$C_{21}H_{22}O_9$	551-15-5	[20, 22]
2	Flower	Herbacetin-3,8-diglucopyranoside	$C_{27}H_{30}O_{17}$	99224-12-1	[22]
3		4',5-Dihydroxy-3',6,7-trimethoxyflavone	$C_{18}H_{16}O_7$	41365-32-6	[22]
4		Quercetin 3,4-diglucoside	$C_{27}H_{30}O_{17}$	29125-80-2	[22]
5		Myricetin 3-galactopyranoside	$C_{21}H_{20}O_{13}$	15648-86-9	[22]
6		Rutin	$C_{27}H_{30}O_{16}$	153-18-4	[22]
7		Kaempferol-7-O-neohesperidoside	$C_{27}H_{30}O_{15}$	17353-03-6	[22]
8	Flower, root	Kaempferol	$C_{15}H_{10}O_6$	520-18-3	[20, 22]
9	Flower	Kaempferol 3-ss-D-galactoside	$C_{21}H_{20}O_{11}$	23627-87-4	[22]
10		6'-O-Malonylisoquercitrin	$C_{24}H_{22}O_{15}$	96862-01-0	[22]
11		Cyanidin-3-O-glucoside	$C_{21}H_{20}O_{11}$	7084-24-4	[22]
12		Isorhamnetin 3-O-glucoside	$C_{22}H_{22}O_{12}$	5041-82-7	[22]
13		Tamarixetin	$C_{16}H_{12}O_7$	603-61-2	[22]
14		Rhamnetin	$C_{16}H_{12}O_7$	90-19-7	[22]
15		Kaempferol 3-O-(3'' ,4''-di-O-acetyl-α-L-rhamnopyranoside)	$C_{25}H_{24}O_{12}$	77307-50-7	[22]
16		Quercetin	$C_{15}H_{10}O_7$	117-39-5	[22]
17		Quercetagetin	$C_{15}H_{10}O_8$	90-18-6	[22]
18		3,5,7-Trihydroxy-4'-methoxyflavone	$C_{16}H_{12}O_6$	491-54-3	[22]
19		Diosmetin-7-O-β-D-glucopyranoside	$C_{22}H_{22}O_{11}$	20126-59-4	[22]

20	root	Luteolin 7-methyl ether	$C_{16}H_{12}O_6$	20243-59-8	[22]
21		complanatuside	$C_{28}H_{32}O_{16}$	116183-66-5	[21]
22		Narcissin	$C_{28}H_{32}O_{16}$	604-80-8	[20]
23		Nicotiflorin	$C_{27}H_{30}O_{15}$	17650-84-9	[20]
24		Apigenin 7-*O*-glucoside	$C_{21}H_{20}O_{10}$	578-74-5	[20]
25		Chrysin	$C_{15}H_{10}O_4$	480-40-0	[20]
26		Apigenin	$C_{15}H_{10}O_5$	520-36-5	[20]
27		Isoliquiritigenin	$C_{15}H_{12}O_4$	961-29-5	[20]
28		Pinostrobin	$C_{16}H_{14}O_4$	480-37-5	[20]
29		Garbanzol	$C_{15}H_{12}O_5$	1226-22-8	[20]
30		Butein	$C_{15}H_{12}O_5$	487-52-5	[20]
31		Methylinissolin 3-O-glucoside	$C_{23}H_{26}O_{10}$	94367-42-7	[20]
32		7-Hydroxy-2-methoxy-4,5-methylenedioxyisoflavane	$C_{17}H_{16}O_5$	77026-91-6	[20]
33		Methylinissolin	$C_{17}H_{16}O_5$	73340-41-7	[20]
34	Flower	Isomucronulatol-7-O-glucoside	$C_{23}H_{28}O_{10}$	94367-43-8	[22]
35		Luteolin-7,3'-di-O-glucoside	$C_{27}H_{30}O_{16}$	52187-80-1	[22]
36		6''-O-Malonylgenistin	$C_{24}H_{22}O_{13}$	51011-05-3	[22]
37		Malonylglycitin	$C_{25}H_{24}O_{13}$	137705-39-6	[22]
38	Flower, root	Calycosin	$C_{16}H_{12}O_5$	20575-57-9	[20-23]
39	Flower	Tectoridin	$C_{22}H_{22}O_{11}$	611-40-5	[22]
40	Flower, root	Formononetin	$C_{16}H_{12}O_4$	485-72-3	[20-23]
41	Flower	Isomucronulatol	$C_{17}H_{18}O_5$	64474-51-7	[22]
42		Biochanin A	$C_{16}H_{12}O_5$	491-80-5	[22]
43	root	calycosin-7-O-β-D-glucoside	$C_{22}H_{22}O_{10}$	20633-67-4	[20, 21, 23]

44	ononin	$C_{22}H_{22}O_9$	486-62-4	[20, 21, 23]
45	calycosin-7-O-β-D-glucoside-6''-O-malonate	$C_{25}H_{24}O_{13}$	-	[23]
46	(6aR,11aR)-3-hydroxy-9,10-dimethoxypter-ocarpan-3-O-β-D-glucoside	$C_{23}H_{26}O_{10}$	94367-42-7	[23]
47	formononetin-7-O-β-D-glucoside-6''-O-malonate	$C_{25}H_{24}O_{12}$	34232-16-1	[23]
48	Astrapterocarpan glucoside-6''-O-malonate	C26H30O13	-	[23]
49	(6aR,11aR)-3-hydroxy-9,10-dimethoxypter-ocarpan	$C_{17}H_{16}O_5$	73340-41-7	[23]
50	(3R)-7,2'-dihydroxy-3',4'-dimethoxyisoflavan	$C_{17}H_{18}O_5$	64474-51-7	[23]
51	Licoagroside D	$C_{22}H_{24}O_{10}$	464196-55-2	[20]
52	Odoratin 7-*O*-glucoside	$C_{23}H_{24}O_{11}$	210413-47-1	[20]
53	Biochanin A 7-*O*-(6-*O*-malonyl-glucoside)	$C_{25}H_{24}O_{13}$	34232-17-2	[20]
54	Pratensein 7-*O*-glucoside	$C_{22}H_{22}O_{11}$	36191-03-4	[20]
55	Isomucronulatol 7-*O*-glucoside	$C_{23}H_{28}O_{10}$	136087-29-1	[20]
56	7-Hydroxy-2'-methoxy-4',5'-methylenedio-xyisoflavane	$C_{17}H_{16}O_5$	77026-91-6	[20]
57	Calycosin 7-*O*-{6''-[-but-2-enoyl]}-glucoside	$C_{26}H_{26}O_{11}$	-	[20]
58	2',8-Dihydroxy-4',7-dimethoxyisoflavane	$C_{17}H_{18}O_5$	-	[20]
59	Odoratin	$C_{17}H_{14}O_6$	53948-00-8	[20]
60	Vesticarpan	$C_{16}H_{14}O_5$	69853-46-9	[20]
61	Astragaluquinone	$C_{17}H_{16}O_6$	158991-20-9	[20]
62	Pratensein	$C_{16}H_{12}O_6$	2284-31-3	[20]
63	Daidzein	$C_{15}H_{10}O_4$	486-66-8	[20]

64		7-Hydroxy-3',5'-dimethoxyisoflavone	$C_{17}H_{14}O_5$	873869-44-4	[20]
65		(3R)-7,29-dihydroxy-3'4'-dimethoxyisoflavan-7-O-β-D-glucoside	$C_{23}H_{28}O_{10}$	-	[23]
66		isoastragaloside I	$C_{45}H_{72}O_{16}$	84676-88-0	[21]
67		isoastragaloside II	$C_{43}H_{70}O_{15}$	86764-11-6	[21]
68		isoastragaloside IV	$C_{41}H_{68}O_{14}$	136033-55-1	[21]
69		astragaloside I	$C_{45}H_{72}O_{16}$	84680-75-1	[21]
70		astragaloside II	$C_{43}H_{70}O_{15}$	84676-89-1	[21]
71		astragaloside III	$C_{41}H_{68}O_{14}$	84687-42-3	[21]
72	root	astragaloside IV	$C_{41}H_{68}O_{14}$	84687-43-4	[21]
73		cyclocephaloside II	$C_{43}H_{70}O_{15}$	215776-78-6	[21]
74		β-D-Glucopyranoside, (3β,6α,16β,20R,24S)-3-[(3,4-di-O-acetyl-β-D-xylopyranosyl)oxy]-20, 24-epoxy-16,25-dihydroxy-9,19-cyclolano-stan-6-yl	$C_{45}H_{72}O_{16}$	1324005-51-7	[21]
75	Flower	Hovetrichoside C	$C_{21}H_{22}O_{11}$	210050-28-5	[22]
76		Prim-O-glucosylcimifugin	$C_{22}H_{28}O_{11}$	80681-45-4	[22]
77	root	3',6-Dihydroxy-4'-methoxyaurone	$C_{16}H_{12}O_5$	32396-80-8	[20]

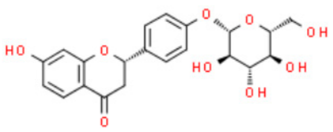

1

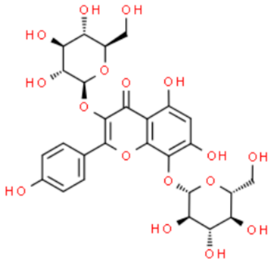

2

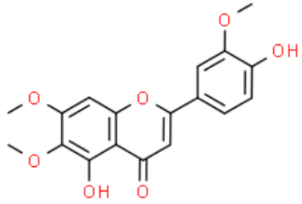

3

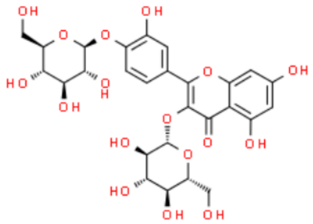

4

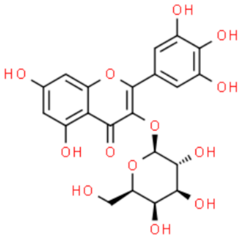

5

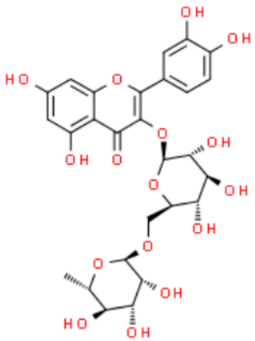

6

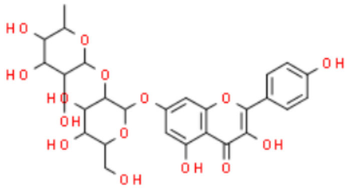

7

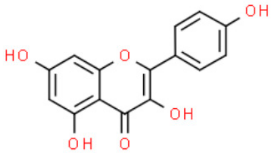

8

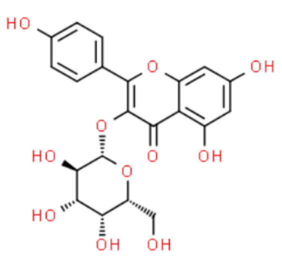

9

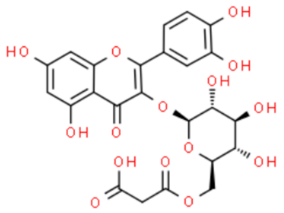

10

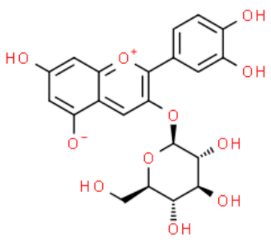

11

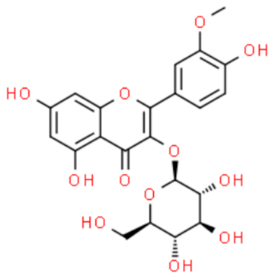

12

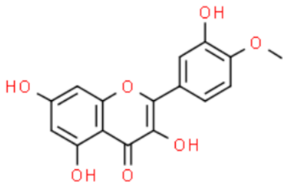

13

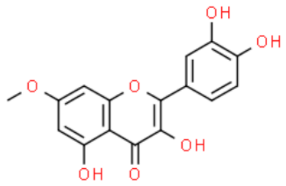

14

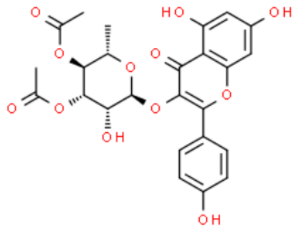

15

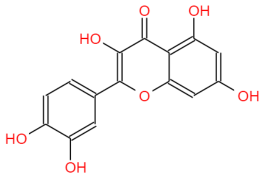

16

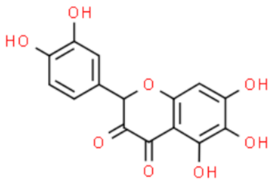

17

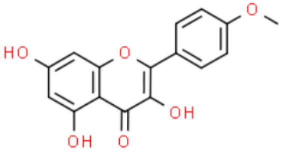

18

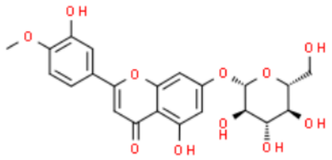

19

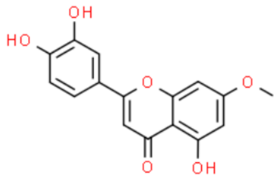

20

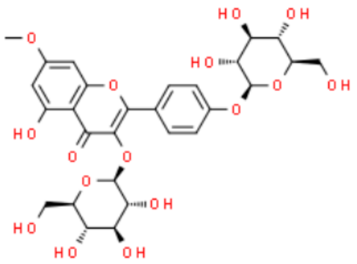

21

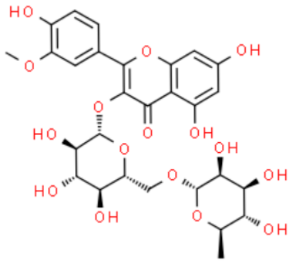

22

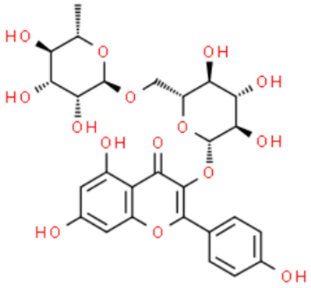

23

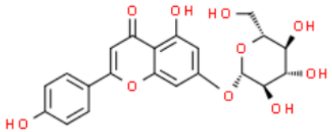

24

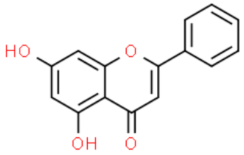

25

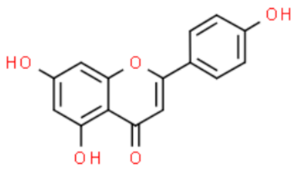

26

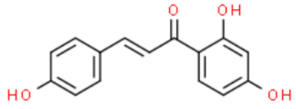

27

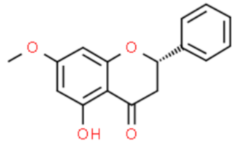

28

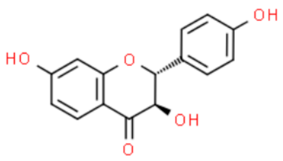

29

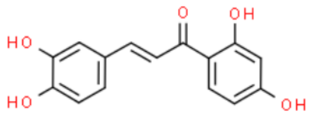

30

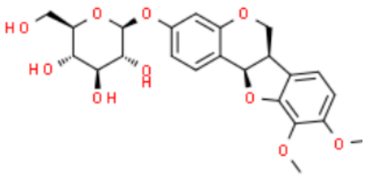

31

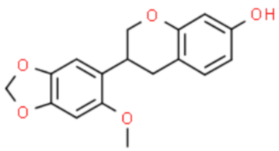

32

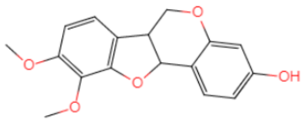

33

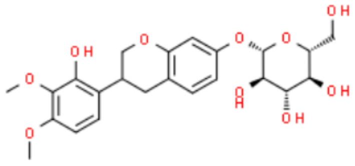

34

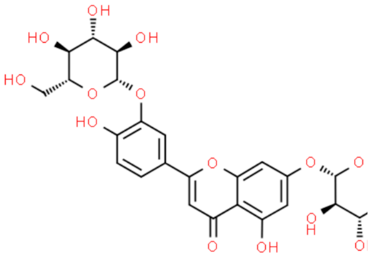

35

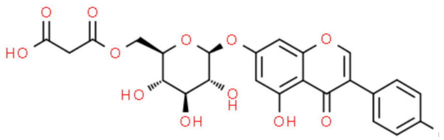

36

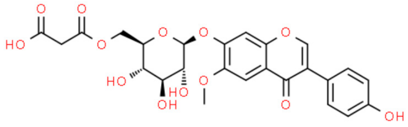

37

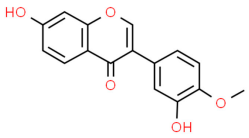

38

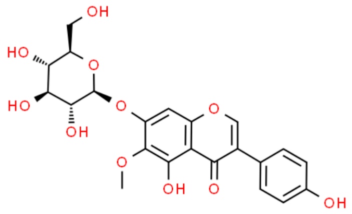

39

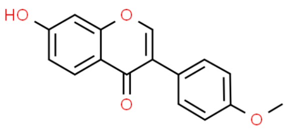

40

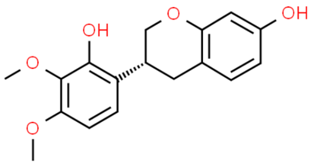

41

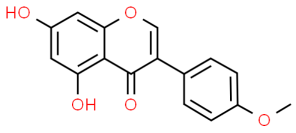

42

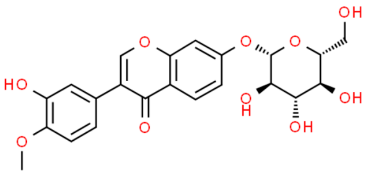

43

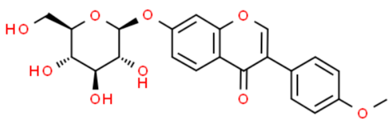

44

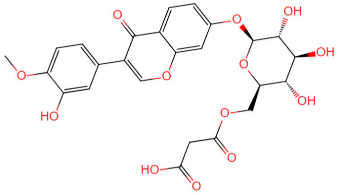

45

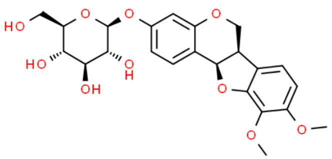

46

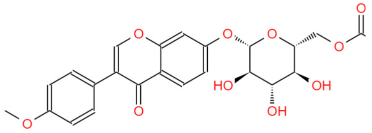

47

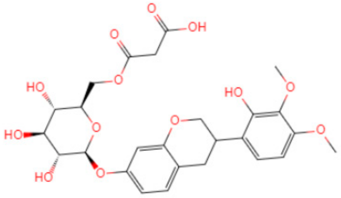

48

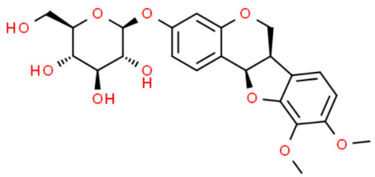

49

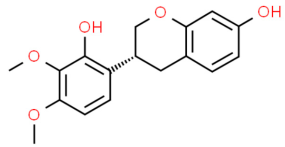

50

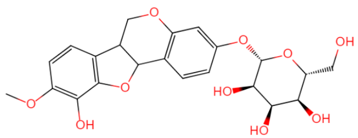

51

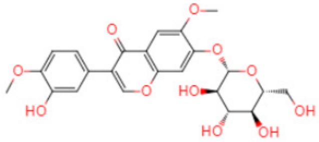

52

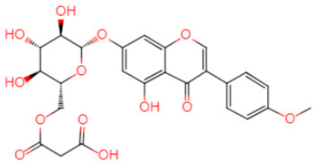

53

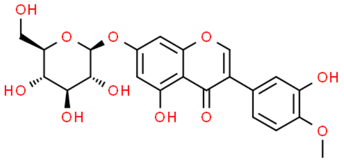

54

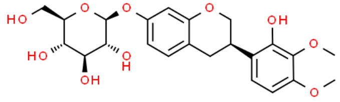

55

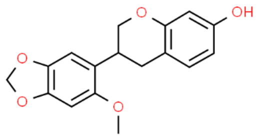

56

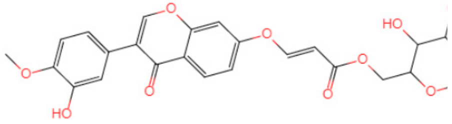

57

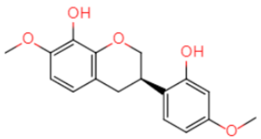

58

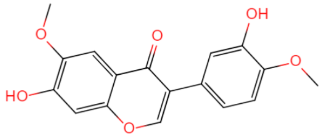

59

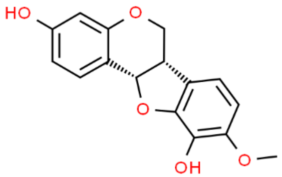

60

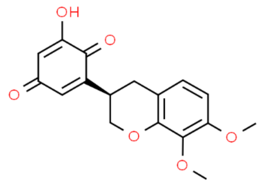

61

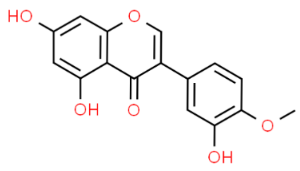

62

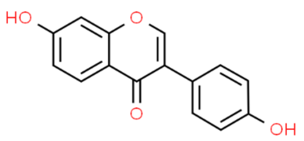

63

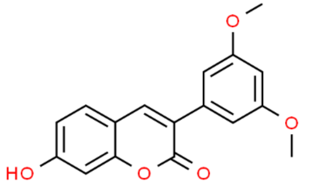

64

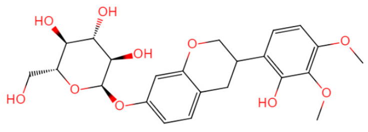

65

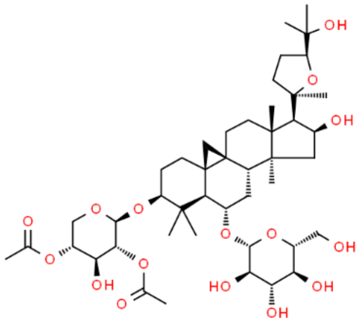

66

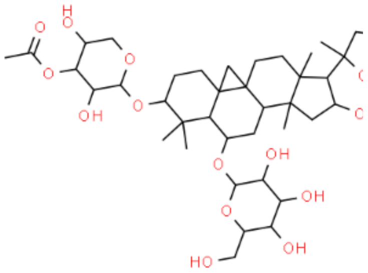

67

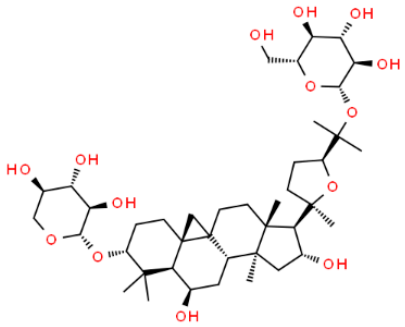

68

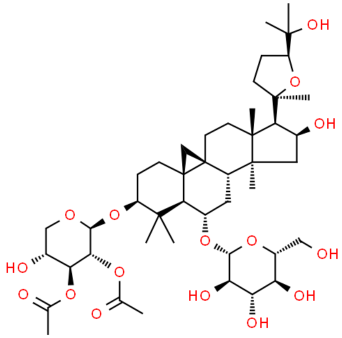

69

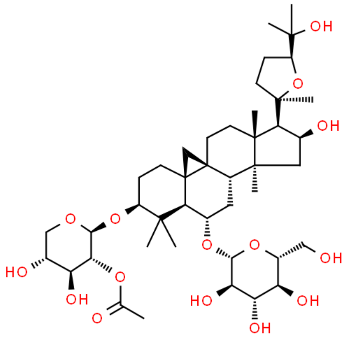

70

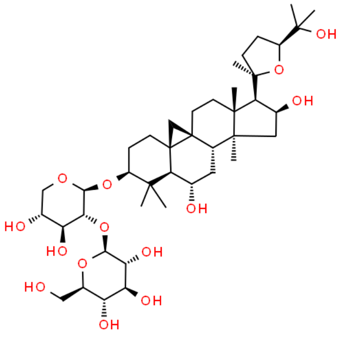

71

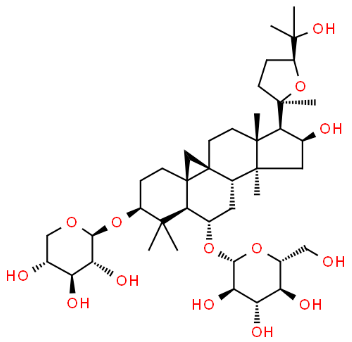

72

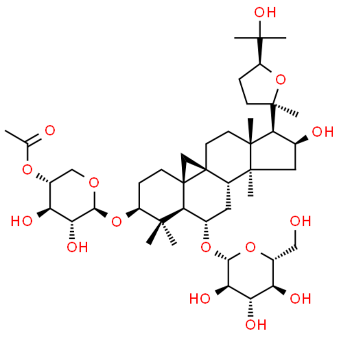

73

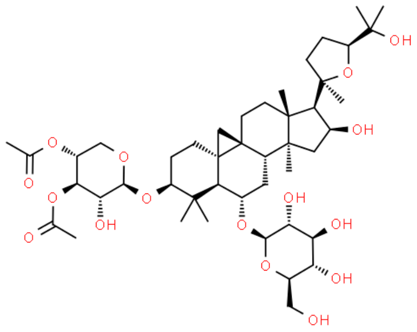

74

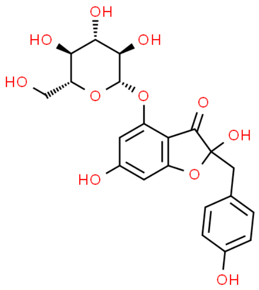

75

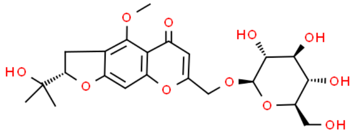

76

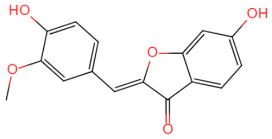

77

Figure S1. Chemical structure of substance in *Astragalus membranaceus*.
